# Clinical Characteristics, Risk Factors, and Outcomes of Patients With Myocardial Injury due to *Klebsiella pneumoniae* Bloodstream Infections

**DOI:** 10.1155/cjid/1795084

**Published:** 2025-01-31

**Authors:** Qingqing Chen, Panpan Xu, Zhihui Guan, Feizhen Song, Xinhua Luo, Xijiang Zhang, Chuming Zhang, Ronghai Lin, Cheng Zheng

**Affiliations:** ^1^Department of Rehabilitation Center, Taizhou Hospital of Zhejiang Province Affiliated to Wenzhou Medical University, Taizhou 318000, China; ^2^Department of Critical Care Medicine, Municipal Hospital Affiliated to Taizhou University, Taizhou 318000, Zhejiang, China; ^3^Department of Emergency, Suzhou Dushuhu Public Hospital (Dushuhu Public Hospital Affiliated to Soochow University), Suzhou 215000, Jiangsu, China; ^4^Department of Critical Care Medicine, Taizhou First People's Hospital, Taizhou 318000, Zhejiang, China; ^5^Department of Critical Care Medicine, Second Affiliated Hospital, Zhejiang University School of Medicine, Hangzhou 310009, Zhejiang, China; ^6^Department of Critical Care Medicine, Shengzhou People's Hospital, Shaoxing 312000, Zhejiang, China; ^7^Department of Clinical Laboratory Medicine, Municipal Hospital Affiliated to Taizhou University, Taizhou, Zhejiang 318000, China

**Keywords:** clinical characteristics, *Klebsiella pneumoniae*, mortality, myocardial injury, risk factors

## Abstract

**Background:** Very few studies have characterized patients with myocardial injury due to *Klebsiella pneumoniae* bloodstream infections (KP-BSI). Our study aimed to investigate the clinical characteristics, risk factors and outcomes of patients with myocardial injury due to KP-BSI.

**Methods:** A double-center retrospective cohort study of patients with KP-BSI was conducted from January 1, 2013 to December 31, 2022. The clinical data was collected by reviewing electronic medical records. Classification of patients with KP-BSI into myocardial injury and nonmyocardial injury groups based on the levels of high-sensitivity cardiac troponin I (hs-cTnI) after 48 h onset of KP-BSI.

**Results:** Patients with myocardial injury due to KP-BSI were generally younger than those without such injuries, with the former presenting a median age of 60 versus 67 in the latter (*p* < 0.001). Conditions like chronic cardiac insufficiency and chronic pulmonary disease were more prevalent in the myocardial injury cohort (10.0% and 7.1%, respectively) compared to those without myocardial injury (4.7% and 2.6%, respectively; *p* values 0.002 and 0.001). However, the nonmyocardial injury group had a higher incidence of solid tumors (15.3% vs. 10.4%, *p*=0.038). Severity assessments like the acute physiology and chronic health evaluation (APACHE) II, the sequential organ failure assessment (SOFA), and the Charlson Comorbidity Index (CCI) all registered higher for the myocardial injury group (all *p* < 0.001). Similarly, intensive care unit (ICU) admissions, use of mechanical ventilation, and central venous catheter (CVC) placement were notably more common in this group (all *p* < 0.001). Regarding infection sources, the myocardial injury group had a higher incidence of pneumonia as the cause for KP-BSI (29.8% vs. 15.9%, *p* < 0.001), whereas liver and biliary tract infections were less frequent compared to their counterparts. Mortality rates at 7, 14, and 28 days, along with in-hospital mortality, were significantly higher for those with myocardial injury (all *p* < 0.001). Multivariate analysis identified age > 67 [adjusted odds ratio (aOR), 2.32; 95% confidence interval (CI), 1.59–3.38], SOFA score > 6 (aOR, 3.04; 95% CI, 2.10–4.39), mechanical ventilation (aOR, 1.67; 95% CI, 1.15–2.39), and CVC in place (aOR, 1.50; 95% CI, 0.96–2.02) as independent prognostic factors for myocardial injury in KP-BSI.

**Conclusions:** Older age (> 67 years), higher SOFA score (> 6), mechanical ventilation, and CVC in place were found to be significantly associated with an increased risk of myocardial injury. Clinical physicians should be alert to the potential for myocardial injury in elderly critically ill patients, especially those who are on mechanical ventilation and have indwelling CVC, in the event of KP-BSI.

## 1. Introduction

Bloodstream infections (BSIs) are a significant global health concern due to their high morbidity and mortality rates. These infections occur when pathogenic microorganisms invade the bloodstream, potentially leading to severe complications such as septic shock, diffuse intravascular coagulation, multiorgan failure, and even death [1–3]. Within China, nosocomial (hospital-acquired) infections are a major source of BSIs, with *Klebsiella pneumoniae* (KP) identified as the second most common Gram-negative pathogen after *Escherichia coli* [4, 5]. A multicenter retrospective study indicates that KP accounts for 20.5% of BSIs, highlighting its prominence in healthcare settings, including our institution [6].

The clinical significance of KP extends beyond its prevalence. It is particularly concerning due to its increasing antibiotic resistance, which complicates the management of BSIs and heightens the risk of severe outcomes, including sepsis and organ dysfunction.

Myocardial injury associated with BSIs has gained increasing attention among clinicians [7–9]. Epidemiological studies estimate that myocardial injury occurs in 20%–50% of BSIs cases, with morbidity and mortality rates in critically ill patients reaching as high as 70%–90% [10, 11]. Despite this, there is a notable gap in the literature regarding the specific pathogens causing myocardial injury in the context of BSIs. As KP develops drug resistance, it not only becomes more challenging to treat but also poses a broader threat to the general population. This resistance can lead to dysregulated immune responses, the production of inflammation-related factors, and critical damage to cardiac cells, especially in compromised individuals [12]. Moreover, KP is known to cause myocardial injury, a common complication in BSIs, which can significantly impact patient outcomes [13]. Given the urgency of understanding the clinical characteristics of myocardial injury associated with KP-BSI, identifying risk factors is crucial. This knowledge can inform strategies to prevent myocardial injury, reduce mortality rates, and improve patient outcomes. Therefore, our focus should encompass not only treating KP-BSIs but also understanding and mitigating its cardiac implications.

## 2. Methods

### 2.1. Patients and Study Design

This double-center retrospective cohort study was conducted in The Second Affiliated Hospital, Zhejiang University School of Medicine, and Municipal Hospital Affiliate to Taizhou University. The study was approved by the Ethics Committee of The Second Affiliated Hospital, Zhejiang University School of Medicine (NO.2019-119) and the Ethics Committee of Municipal Hospital Affiliate to Taizhou University (No.2021 KTWST002). The ethical committee stated that informed consent was not required for this retrospective observational study. Furthermore, a statement of permission from patients for submission was not needed, as no personal information was included.

All KP strains isolated from blood cultures obtained from adult patients who were admitted between July 2013 and June 2022 were entered in our database. Clinical and laboratory data were extracted from the electronic medical record for each patient encounter. Patients were categorized into two groups (myocardial injury vs. nonmyocardial injury) based on the levels of high-sensitivity cardiac troponin I (hs-cTnI) after 48h onset of KP-BSI. Patients could only be included in the study once, even if they had more than two positive blood cultures during the study period. Exclusion criteria: (1) Patients with age < 18 years old; (2) the medical record was missing or incomplete (data less than 70%); (3) Patients who died or were discharged within 72 h after onset of BSIs.

### 2.2. Data Collection

We collected basic information about patients, such as their age, sex, prior hospital days, prior healthcare interventions (within 30 days before the onset of KP-BSI), hospitalization ward at BSI onset, sources of infection, from their medical records. Patient Comorbidity was determined using the Charlson Comorbidity Index (CCI, used to predict 10-year mortality in patients who may have a range of comorbidities) [14]. Furthermore, the biological indicators including blood routine test, liver function, serum creatinine, procalcitonin (which helps differentiate between bacterial and viral infections and can be used to monitor the severity of sepsis and the response to antibiotic treatment) [15], C-reactive protein (which is a nonspecific marker of inflammation and can be used to monitor the body's response to various inflammatory conditions) at the onset of BSI were recorded. We also measured specific markers related to heart health after the onset of KP-BSI, such as myocardial zymogram (used to diagnose myocardial infarction and other forms of myocardial injury), hs-cTnI (a highly sensitive and specific biomarker for myocardial injury), NT-proBNP (a valuable biomarker for the diagnosis and monitoring of heart failure). We used several scores to understand how severe the illness was, including the acute physiology and chronic health evaluation (APACHE) II score, Pitt bacteremia score, and sequential organ failure assessment (SOFA) score. These scores help predict the risk of death and the level of organ dysfunction in critically ill patients. Finally, we looked at the outcomes of the patients, such as how long they stayed in the hospital or ICU, how long they needed mechanical ventilation, and whether they survived 14 or 28 days after the infection started.

### 2.3. Microbiological Analysis

Blood cultures were performed using aerobic bottles in the BacT/ALERT 3D automated system (Becton-Dickinson, Sparks, MD, USA) and isolates identified using standard procedures. All KP were routinely tested for antimicrobial susceptibility using the Vitek 2 system (bioMerieux, France) or Kirby–Bauer Disk Diffusion method (Oxioid, UK) performed on Mueller–Hinton agar (BD, Franklin Lakes, NJ). All results were interpreted according to the Clinical and Laboratory Standards Institute (CLSI, 2017).

### 2.4. Definitions

The Center for Disease Control (CDC) definition for BSI to determine the clinical significance of KP was used [16]. The diagnosis of myocardial injury aligned with the Fourth Universal definition of Myocardial Infarction [17]. Cardiac troponin testing was performed after onset of KP-BSI and was repeated 24 or 48 h after the onset of symptoms at the discretion of the attending physician in accordance with guidelines. Hs-cTnI was measured in hospitalized patients using the Abbott Architect STAT hs-cTnI assay (sex-specific > 99th percentile upper reference limit: female: > 16 ng/L, male: > 34 ng/L). Myocardial injury due to KP-BSI can be defined as an increase in hs-cTnI 48 h after the onset of KP-BSI.

### 2.5. Statistical Analysis

Statistical analyses and mapping were performed with SPSS for Windows (version 26.0; SPSS, IBM Corporation, Armonk, NY, USA) and GraphPad Prism 9.5 (GraphPad Software, La Jolla, CA, USA). The characterization of the data were appropriately tailored to the nature of each variable: categorical variables were summarized by frequencies and percentages (*n* (%)), normally distributed continuous variables by means and standard deviations (mean (SD)), and non-normally distributed continuous variables by medians and interquartile ranges (median (IQR)) or ranges (median (range)) as dictated by the distribution characteristics.

To compare groups, the Student's *t*-test was employed for normally distributed continuous variables, while the Mann–Whitney *U*-test was utilized for those not following a normal distribution. For categorical data, the Pearson *χ*^2^ test was chosen to determine statistical significance between groups. When continuous variables required dichotomization for the analyses, they were converted and analyzed as categorical variables. Collinearity analysis demonstrated no collinearity among the variables. Multivariate analysis was performed using variables associated with the outcome in univariate analysis at a *p* value of < 0.05. Odds ratios (OR) and 95% confidence intervals (CI) were estimated from logistic regression analysis. Survival curves were estimated by the Kaplan–Meier product-limit method, and survival distributions between groups were compared using the log-rank test. All tests were two-tailed, and *p* values < 0.05 were considered statistically significant.

## 3. Results

### 3.1. Demographic and Clinical Characteristics

**Figure 1:**
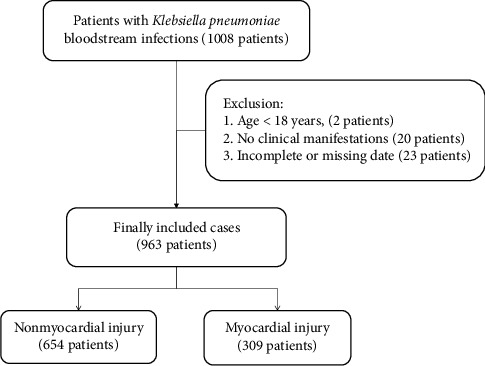
Flowchart of study participant enrolment.

Over a period of 9 years, a total of 963 patients were included in the study after excluding 2 patients under 18 years old, 20 patients with no clinical manifestations, and 23 patients with incomplete or missing data. Among the 963 patients, 309 (32.1%) patients had comorbid myocardial injury and 654 (67.9%) patients had no comorbid myocardial injury (Figure 1).

The demographic characteristics of the patients included in this study are comprehensively summarized in Table 1. The patients diagnosed with KP-BSI had a median age of 62 years, with the majority being male (68.7%, 662/963). A noteworthy observation is that patients who developed myocardial injury were generally younger compared to their counterparts without myocardial injury [median age (IQR): 60 years (50–69) versus 67 years (53–77); *p* < 0.001]. However, the distribution of gender between the two groups did not show significant variation. Comorbid conditions were prevalent among the patient population, with diabetes (19.1%) and cerebrovascular accidents (17.9%) being the most commonly reported. It was found that chronic cardiac insufficiency and chronic pulmonary disease (including chronic obstructive pulmonary disease or severe asthma) were more frequently present in patients with myocardial injury (10.0% vs. 4.7%, *p*=0.002 and 7.1% vs. 2.6%, *p*=0.001, respectively). Conversely, the presence of solid tumors was more prevalent among patients without myocardial injury (10.4% vs. 15.3%, *p*=0.038). In terms of severity indices, the myocardial injury group exhibited higher scores on the APACHE II [median (IQR): 17 (12–24) versus 13 (10–18); *p* < 0.001], the SOFA [median (IQR): 8 (5–12) versus 4 (2–6); *p* < 0.001], and the CCI [median (IQR): 4 (2–6) versus 3 (1–5); *p* < 0.001]. Furthermore, admission rates to the ICU were significantly higher for patients with myocardial injury (75.1% vs. 42.2%, *p* < 0.001). This group also had a greater reliance on mechanical ventilation (35.0% vs. 17.1%, *p* < 0.001) and a higher incidence of central venous catheter (CVC) placement (72.1% vs. 49.1%, *p* < 0.001).

### 3.2. Biological Indicators

**Figure 2:**
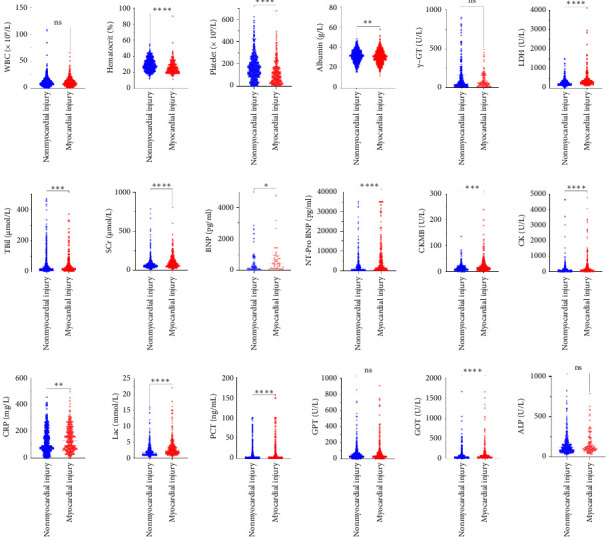
Scatter plots of biological indicators in patients with nonmyocardial injury and myocardial injury group. γ-GT, gamma glutamyl transpeptidase; ALP, alkaline phosphatase; BNP, brain natriuretic peptide; CK, creatine kinase; CK-MB, creatine kinase isoenzymes; CRP, C-reactive protein; GOT, glutamic-oxaloacetic transaminase; GPT, glutamic-pyruvic transaminase; LDH, lactic dehydrogenase; NT-pro BNP, N terminal pro B type natriuretic peptide; PCT, procalcitonin; SCr, serum creatinine; TBil, total bilirubin; WBC, white blood count.

Compared with the nonmyocardial injury group, hematocrit, platelet count, and albumin were lower in the myocardial injury group, whereas glutamic-oxaloacetic transaminase (GOT), lactic dehydrogenase (LDH), total bilirubin (TBil), serum creatinine, B-type natriuretic peptide (BNP), NT-pro BNP, creatine kinase isoenzyme MB, creatine kinase, lactic acid, C-reactive protein, and procalcitonin were significantly higher than those in the nonmyocardial injury group (all *p* < 0.05) (Figure 2).

### 3.3. Sources of KP-BSI

**Figure 3:**
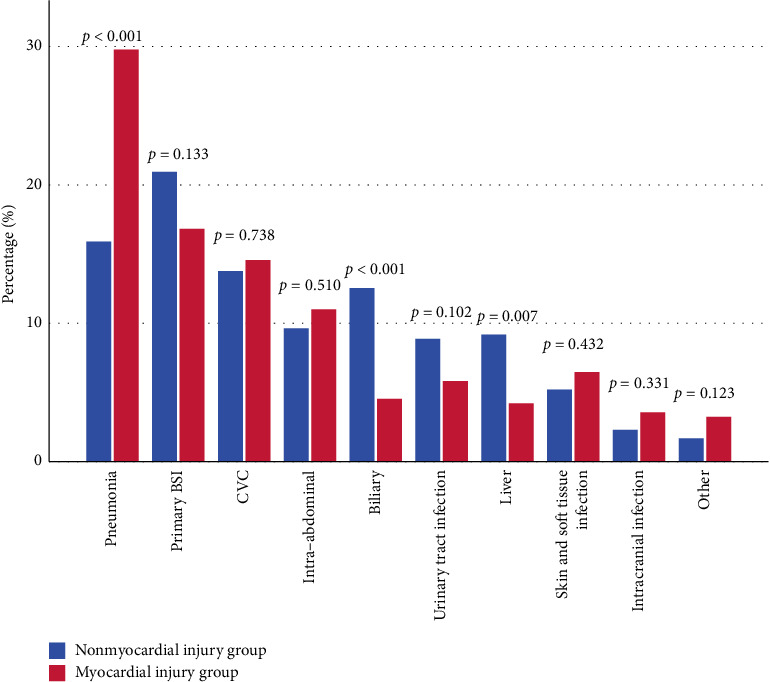
The source of *K. pneumoniae* BSI. Other: mediastinum, bone and joint, endocarditis. BSI, bloodstream infection; CVC, central venous catheter.

Figure 3 elucidates the predominant sources of KP-BSI among the patient cohort. The data highlight pneumonia as the leading cause followed closely by primary BSI. A comparative analysis revealed that in the group of patients who experienced myocardial injury, the incidence of pneumonia as a source of KP-BSI was significantly higher (*p* < 0.001) compared to the nonmyocardial injury group. Conversely, the myocardial injury cohort exhibited a lower prevalence of liver infections (*p*=0.007) and biliary tract infections (*p* < 0.001) as sources of KP-BSI.

### 3.4. Outcomes

**Figure 4:**
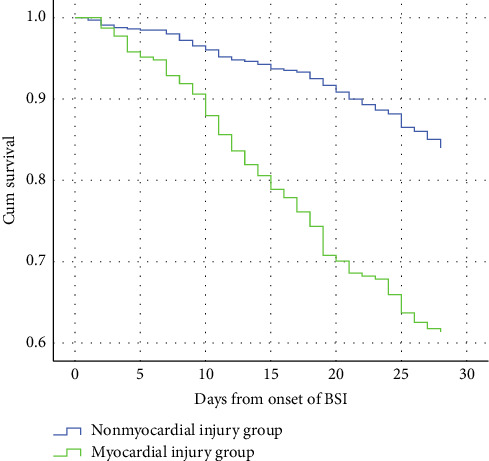
Kaplan–Meier estimates of survival in patients with myocardial injury due to *Klebsiella pneumoniae* bloodstream infections.

Table 2 presents a stark comparison in mortality rates at various time points for patients with KP-BSIs, stratified by the presence or absence of myocardial injury. The data reveal that the 7-day, 14-day, 28-day, and in-hospital mortality rates were all significantly higher in the myocardial injury group compared to those without myocardial injury (all *p* < 0.001), which was consistent with the survival curves of the patients in both the groups (Figure 4). Despite the clear disparity in survival outcomes, an interesting finding is that the duration of hospital stays, the length of stay in the ICU and days of mechanical ventilation after KP-BSI onset did not differ significantly between the two groups.

### 3.5. Independent Prognostic Factors for Myocardial Injury due to KP-BSI

**Figure 5:**
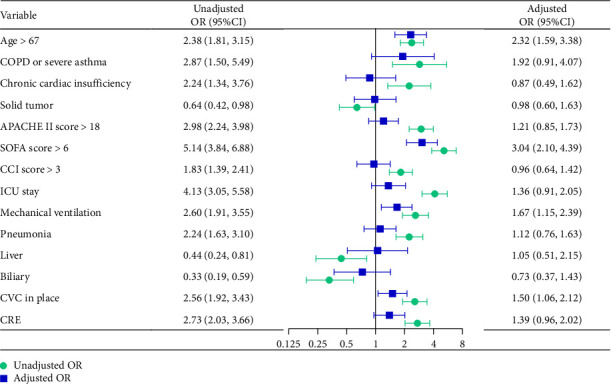
Forest plot of the results of the univariate and multivariable logistic regression analyses for prognostic factors for patient with myocardial injury due to *Klebsiella pneumoniae* bloodstream infections.

We converted age, APACHE II score, SOFA score, and CCI score into dichotomous variables based on the Jordon Index, and constructed a stepwise logistic regression multivariable model by combining the above variables with the count variables that had significance at a *p* < 0.05 level in the univariate analysis. The variance inflation factor (VIF) values of all variables in the model were less than 3. The multivariate logistic regression model showed that the independent prognostic factors for myocardial injury due to KP-BSI were age > 67 years [adjusted OR (aOR), 2.32; 95% CI, 1.59–3.38], SOFA score > 6 (aOR, 3.04; 95% CI, 2.10–4.39), mechanical ventilation (aOR, 1.67; 95% CI, 1.15–2.39), and CVC in place (aOR, 1.50; 95% CI, 0.96–2.02) (Figure 5).

## 4. Discussion

Our study is currently the most extensive clinical investigation into myocardial injury precipitated by KP-BSI, providing valuable insights into the epidemiology and prognosis of this condition. The findings of this research are multifaceted: Firstly, myocardial injury is a relatively common consequence of KP-BSI, affecting approximately 32.1% of cases. This rate is non-negligible and warrants clinical attention, considering the significant impact on patient outcomes. Secondly, the prognosis for patients suffering from myocardial injury due to KP-BSI is notably poor. This is reflected in the escalated mortality rates observed at 7, 14, and 28 days, as well as during the entire duration of hospitalization. Thirdly, we have identified several prognostic indicators that are linked to an increased risk of myocardial injury in KP-BSI patients. As detailed in Table 1 and illustrated in Figure 5, factors such as age exceeding 67 years, a SOFA score greater than 6, the requirement of mechanical ventilation, and the presence of a CVC were found to be independent predictive factors for myocardial injury in this patient population.

### 4.1. Epidemiology and Mechanism of Myocardial Injury Caused by KP-BSI

Our research corroborates findings from previous studies, such as the 13.8% incidence of stress-induced cardiomyopathy in patients with sepsis and the high prevalence (84%) of elevated cardiac hs-cTnI levels in critically ill patients in the ICU with BSI [18]. Another study of BSI-related myocardial injury in ICU patients showed that 84 per cent of critically ill patients had a raised hs-cTnI level at some stage during their stay in the ICU [19]. These figures parallel the cardiac complications observed in patients with COVID-19, where myocardial injury was noted in 7.2% of hospitalized patients and in 22% of those admitted to the ICU [20]. Subsequent early research from China reported acute myocardial injury in 12% of all patients and 31% of those in the ICU [21]. The pathogenesis of myocardial injury related to KP-BSI could involve various factors, including structural components of the bacteria. While some studies suggest a role for capsular polysaccharides and lipopolysaccharides in myocardial injury risk, further research is needed to fully understand these mechanisms [22]. Additionally, an immune response to the pathogen might play a role, but the exact nature of this involvement requires more investigations [23]. Given these insights, it is important for clinicians to be aware of the potential for cardiac dysfunction in patients with KP-BSI. Early detection and appropriate intervention could be beneficial for patient outcomes.

### 4.2. Mortality Rate and Multifactorial Causes in KP-BSI Patients

Our investigation has uncovered a notably elevated mortality rate among patients with myocardial injury concurrent with KP-BSI, which appears to be multifactorial: (1) Myocardial injury stemming from KP-BSI can precipitate heart failure, characterized by the heart's diminished efficiency in pumping blood, which in turn may result in inadequate systemic organ oxygenation and an escalated mortality risk [24]. (2) Severe infection responses to KP-BSIs are capable of inducing multiorgan failure, exacerbating the patient's clinical status and further elevating the likelihood of death [25]. (3) KP-BSI frequently afflicts individuals with pre-existing health conditions or compromised immune systems [26, 27]. KP-BSI frequently afflicts individuals with pre-existing health conditions or compromised immune systems. Notably, our study indicates a significantly higher prevalence of chronic cardiac insufficiency in the myocardial injury cohort compared to those without myocardial injury. Such underlying health issues and immune dysfunctions can amplify infection severity and, consequently, the mortality risk. These findings suggest that comprehensive patient management strategies should not only address the immediate infectious process of KP-BSI but also consider the broader implications of myocardial injury, particularly in patients with pre-existing cardiac conditions or immunocompromised states, to mitigate the associated increased risk of death.

### 4.3. Clinical Implications and Management Strategies for KP-BSI

In our analysis, a correlation was observed between high SOFA scores in older patients and an elevated incidence of severe comorbidities and multiorgan dysfunction. Such clinical scenarios can be complex and may have implications for cardiac function, although the exact nature of this relationship requires further investigation. Furthermore, critical care interventions, such as mechanical ventilation and the utilization of CVC, while essential, also carry risks. Mechanical ventilation, for instance, has been associated with ventilator-associated pneumonia, which could potentially facilitate the entry of KP into the bloodstream, leading to myocardial compromise [28–31]. Similarly, CVC, though often necessary, can be a nidus for BSIs, further increasing the likelihood of cardiac involvement [32]. Given these insights, it is imperative for clinicians to exercise heightened caution for myocardial injury during the management of KP-BSI, particularly in elderly patients or those with a compromised baseline health status. Monitoring for cardiac complications should be considered as part of a comprehensive therapeutic strategy for these patients, with the aim of improving clinical outcomes. The above results highlight the necessity of early intervention, such as how to develop effective measures to remove endotracheal intubation as early as possible, and how to timely remove nonessential CVC, which may avoid the occurrence of myocardial injury due to KP-BSI.

## 5. Limitations

There are several limitations to this study, including those inherent to a retrospective observational study design. This study contributes to the understanding of prognostic factors for myocardial injury in patients with KP-BSI. However, there are some limitations to be acknowledged. First, the study was retrospective and conducted at two centers, which may limit the generalizability of the findings and the results are not necessarily generalizable to other hospitals. Future studies with larger sample sizes and multicenter designs are warranted to validate our results. Second, the retrospective cohort design limits the ability to establish causal relationships. In the future, prospective study designs will be more robust in terms of being able to establish a causal relationship between KP-BSI and myocardial injury. It is recommended that future research focus on the role of specific biomarkers in the evaluation of myocardial injure. Third, the decision to include only patients with complete data might introduce selection bias, as those with incomplete data could have systematically different characteristics.

## 6. Conclusions

Older age (> 67 years), higher SOFA score (> 6), mechanical ventilation, and CVC in place were found to be significantly associated with an increased risk of myocardial injury. Clinical physicians should be alert to the potential for myocardial injury in elderly critically ill patients, especially those who are on mechanical ventilation and have indwelling CVCs, in the event of KP-BSI. Further research is needed to elucidate the pathophysiological processes by which KP leads to myocardial injury, which could inform the development of novel therapeutic strategies.

## Figures and Tables

**Table 1:** Baseline characteristics of patients with *Klebsiella pneumoniae* bloodstream infection.

Characteristics	Total (*n* = 963)	Myocardial injury (*n* = 309)	Nonmyocardial injury (*n* = 654)	Odds ratios	*p* value
Age, median years (IQR)	62 (51, 73)	60 (50, 69)	67 (53, 77)	1.021	**< 0.000**
Male sex	662 (68.7%)	204 (66.0%)	458 (70.0%)	0.831	0.210
Co-morbidities					
Trauma	125 (13.0%)	45 (14.6%)	80 (12.2%)	0.818	0.315
Diabetes mellitus	184 (19.1%)	62 (20.1%)	122 (18.7%)	0.914	0.603
Solid tumor	132 (13.7%)	32 (10.4%)	100 (15.3%)	0.640	**0.038**
Cerebrovascular accident	172 (17.9%)	53 (17.2%)	119 (18.2%)	1.074	0.693
Chronic kidney disease	22 (2.3%)	9 (2.9%)	13 (2.0%)	1.479	0.370
Chronic cardiac insufficiency	62 (6.4%)	31 (10.0%)	31 (4.7%)	2.241	**0.002**
Chronic liver disease	53 (5.5%)	15 (4.9%)	38 (5.8%)	1.029	0.138
COPD or severe asthma	37 (4.0%)	22 (7.1%)	17 (2.6%)	2.872	**0.001**
APACHE II score, median (IQR)	14 (10, 20)	17 (12, 24)	13 (10, 18)	1.086	**< 0.001**
SOFA score, median (IQR)	5 (3, 8)	8 (5, 12)	4 (2, 6)	1.214	**< 0.001**
CCI, median (IQR)	3 (2, 6)	4 (2, 6)	3 (1, 5)	1.086	**< 0.001**
Hospitalization ward					
ICU stay	508 (52.8%)	232 (75.1%)	276 (42.2%)	4.126	**< 0.001**
Clinical information within 48 h before blood sampling					
Surgical procedure	488 (50.7%)	164 (53.1%)	324 (49.5%)	1.152	0.306
CVC in place	541 (56.2%)	220 (71.2%)	321 (49.1%)	2.564	**< 0.001**
Mechanical ventilation	220 (22.8%)	108 (35.0%)	112 (17.1%)	2.600	**< 0.001**
Nosocomial infection	752 (78.1%)	270 (87.4%)	482 (73.7%)	0.405	0.618

*Note:* Bold indicates statistical differences.

Abbreviations: APACHE, acute physiology and chronic health evaluation; CCI, Charlson Comorbidity Index; COPD, chronic obstructive pulmonary disorder; CVC, central venous catheter; ICU, intensive care unit; IQR, interquartile range; SOFA, sequential organ failure assessment.

**Table 2:** Comparison of outcome between nonmyocardial injury group and myocardial injury group.

Prognostic indicators	Total (*n* = 963)	Myocardial injury (*n* = 309)	Nonmyocardial injury (*n* = 654)	*p* value
Total hospitalization days (IQR)	27 (14, 52)	28 (14, 54.5)	27 (14.75, 51.00)	0.908
Total ICU residence days (IQR)	20 (9, 40.25)	19 (8.25, 34)	22 (9, 44.25)	0.083
Days of mechanical ventilation after BSI onset (IQR)	9 (4, 28)	11 (4, 31.5)	8 (3, 26)	0.370
7 days total mortality rate (*n*, %)	215 (22.3%)	122 (39.5%)	93 (9.7%)	**< 0.001**
14 days total mortality rate (*n*, %)	275 (28.5%)	153 (49.5%)	122 (18.7%)	**< 0.001**
28 days total mortality rate (*n*, %)	316 (32.8%)	171 (55.3%)	145 (22.2%)	**< 0.001**
In-hospital mortality (*n*, %)	366 (38.0%)	193 (62.5%)	173 (26.5%)	**< 0.001**

*Note:* Bold indicates statistical differences.

Abbreviation: IQR, interquartile range.

## Data Availability

The data used and/or analyzed in this study are available from the corresponding author on reasonable request.

## Nomenclature

KP-BSI: *Klebsiella pneumoniae* bloodstream infections
hs-cTnI: High-sensitivity cardiac troponin I
APACHE II: Acute physiology and chronic health evaluation II
SOFA: Sequential organ failure assessment
CCI: Charlson comorbidity index
ICU: Intensive care unit
VIF: Variance inflation factor
aOR: Adjusted odds ratio
CI: Confidence interval
CVC: Central venous catheter
BSIs: Bloodstream infections;
*K. pneumoniae*: *Klebsiella pneumoniae*
NT-proBNP: N-terminal pro-B type natriuretic peptide
CDC: Center for disease control
SD: Standard deviations
IQR: Interquartile ranges
OR: Odds ratios
GOT: Glutamic-oxaloacetic transaminase
LDH: Lactic dehydrogenase
TBil: Total bilirubin
BNP: B-type natriuretic peptide

## References

[1] Pien B. C., Sundaram P., Raoof N. (2010). The Clinical and Prognostic Importance of Positive Blood Cultures in Adults. *The American Journal of Medicine*.

[2] Goto M., Al-Hasan M. N. (2013). Overall Burden of Bloodstream Infection and Nosocomial Bloodstream Infection in North America and Europe. *Clinical Microbiology and Infection: The Official Publication of the European Society of Clinical Microbiology and Infectious Diseases*.

[3] Holmbom M., Giske C. G., Fredrikson M. (2016). 14-Year Survey in a Swedish County Reveals a Pronounced Increase in Bloodstream Infections (BSI). Comorbidity-An Independent Risk Factor for Both BSI and Mortality. *PLoS One*.

[4] Jiang Z. Q., Wang S. D., Feng D. D., Zhang B. X., Mao S. H., Wu J. N. (2019). Epidemiological Risk Factors for Nosocomial Bloodstream Infections: A Four-Year Retrospective Study in China. *Journal of Critical Care*.

[5] Tian L., Sun Z., Zhang Z. (2018). Antimicrobial Resistance of Pathogens Causing Nosocomial Bloodstream Infection in Hubei Province, China, from 2014 to 2016: A Multicenter Retrospective Study. *BMC Public Health*.

[6] Shi Q., Quan J., Lan P. (2020). Prevalence and Characteristics of Pks Gene Cluster Harbouring *Klebsiella pneumoniae* from Bloodstream Infection in China. *Epidemiology and Infection*.

[7] Pulido J. N., Afessa B., Masaki M. (2012). Clinical Spectrum, Frequency, and Significance of Myocardial Dysfunction in Severe Sepsis and Septic Shock. *Mayo Clinic Proceedings*.

[8] Sevilla Berrios R. A., O’Horo J. C., Velagapudi V., Pulido J. N. (2014). Correlation of Left Ventricular Systolic Dysfunction Determined by Low Ejection Fraction and 30-Day Mortality in Patients With Severe Sepsis and Septic Shock: A Systematic Review and Meta-Analysis. *Journal of Critical Care*.

[9] Fleischmann C., Thomas-Rueddel D. O., Hartmann M. (2016). Hospital Incidence and Mortality Rates of Sepsis. *Deutsches Arzteblatt international*.

[10] Li Y., Ge S., Peng Y., Chen X. (2013). Inflammation and Cardiac Dysfunction During Sepsis, Muscular Dystrophy, and Myocarditis. *Burns & trauma*.

[11] Pan P., Wang X., Liu D. (2018). The Potential Mechanism of Mitochondrial Dysfunction in Septic Cardiomyopathy. *Journal of International Medical Research*.

[12] Alicino C., Giacobbe D. R., Orsi A. (2015). Trends in the Annual Incidence of Carbapenem-Resistant *Klebsiella pneumoniae* Bloodstream Infections: A 8-Year Retrospective Study in a Large Teaching Hospital in Northern Italy. *BMC Infectious Diseases*.

[13] Rudiger A., Singer M. (2007). Mechanisms of Sepsis-Induced Cardiac Dysfunction. *Critical Care Medicine*.

[14] Charlson M. E., Pompei P., Ales K. L., MacKenzie C. R. (1987). A New Method of Classifying Prognostic Comorbidity in Longitudinal Studies: Development and Validation. *Journal of Chronic Diseases*.

[15] Vikse J., Henry B. M., Roy J., Ramakrishnan P. K., Tomaszewski K. A., Walocha J. A. (2015). The Role of Serum Procalcitonin in the Diagnosis of Bacterial Meningitis in Adults: A Systematic Review and Meta-Analysis. *International Journal of Infectious Diseases: IJID: Official Publication of the International Society for Infectious Diseases*.

[16] Bloodstream Infection Event (Central Line-Associated Bloodstream Infection and Non-central Line Associated Bloodstream Infection) National Healthcare Safety Network (NHSN).

[17] Thygesen K., Alpert J. S., Jaffe A. S. (2018). Fourth Universal Definition of Myocardial Infarction (2018). *Journal of the American College of Cardiology*.

[18] Sato R., Kuriyama A., Takada T., Nasu M., Luthe S. K. (2016). Prevalence and Risk Factors of Sepsis-Induced Cardiomyopathy: A Retrospective Cohort Study. *Medicine*.

[19] Lo J., Lei K., Webb I. (2013). Myocardial Injury in Critically Ill Patients Admitted With Noncardiac Diagnoses. *Critical Care*.

[20] Ning Q., Wu D., Wang X. (2022). The Mechanism Underlying Extrapulmonary Complications of the Coronavirus Disease 2019 and its Therapeutic Implication. *Signal Transduction and Targeted Therapy*.

[21] Niraula A., Baral N., Lamsal M., Bataju M., Thapa S. (2022). Potential Role of Biochemical Markers in the Prognosis of COVID-19 Patients. *SAGE Open Medicine*.

[22] Micek S. T., Ward S., Fraser V. J., Kollef M. H. (2004). A Randomized Controlled Trial of an Antibiotic Discontinuation Policy for Clinically Suspected Ventilator-Associated Pneumonia. *Chest*.

[23] Jagielska B., Ozdowska P., Gepner K. (2020). Cardiotoxicity Danger in Immunotherapy. *IUBMB Life*.

[24] Davis J., Sapp J. (2020). The Risk and Prevention of Sudden Death in Patients With Heart Failure With Reduced Ejection Fraction. *Current Opinion in Cardiology*.

[25] Zhou M., Wu R., Dong W., Leong J., Wang P. (2010). Accelerated Apoptosis Contributes to Aging-Related Hyperinflammation in Endotoxemia. *International Journal of Molecular Medicine*.

[26] Dar H. A., Zaheer T., Shehroz M. (2019). Immunoinformatics-Aided Design and Evaluation of a Potential Multi-Epitope Vaccine Against Klebsiella Pneumoniae. *Vaccines*.

[27] El-Mokhtar M. A., Hassanein K. M., Ahmed A. S., Gad G. F., Amin M. M., Hassanein O. F. (2020). Antagonistic Activities of Cell-Free Supernatants of Lactobacilli Against Extended-Spectrum β-Lactamase Producing *Klebsiella pneumoniae* and *Pseudomonas aeruginosa*. *Infection and Drug Resistance*.

[28] Yin Y., Sun M., Li Z. (2022). Exploring the Nursing Factors Related to Ventilator-Associated Pneumonia in the Intensive Care Unit. *Frontiers in Public Health*.

[29] Wang Y. M., Chen Y., Zheng Y. J. (2021). Low Fluid Intake Volume During the First 24 h and Persistent Negative Fluid Balance From the Second Day are Associated With Favorable Prognosis for Patients With Sepsis. *Experimental and Therapeutic Medicine*.

[30] Grønnemose R. B., Garde C., Wassmann C. S. (2021). Bacteria-Host Transcriptional Response During Endothelial Invasion by *Staphylococcus aureus*. *Scientific Reports*.

[31] Husain-Syed F., McCullough P. A., Birk H. W. (2015). Cardio-Pulmonary-Renal Interactions: A Multidisciplinary Approach. *Journal of the American College of Cardiology*.

[32] Khieosanuk K., Fupinwong S., Tosilakul A., Sricharoen N., Sudjaritruk T. (2022). Incidence Rate and Risk Factors of Central Line-Associated Bloodstream Infections Among Neonates and Children Admitted to a Tertiary Care University Hospital. *American Journal of Infection Control*.

